# Novel multi-component nanopharmaceuticals derived from poly(ethylene) glycol, retro-inverso-Tat nonapeptide and saquinavir demonstrate combined anti-HIV effects

**DOI:** 10.1186/1742-6405-3-12

**Published:** 2006-04-24

**Authors:** Li Wan, Xiaoping Zhang, Simi Gunaseelan, Shahriar Pooyan, Olivia Debrah, Michael J Leibowitz, Arnold B Rabson, Stanley Stein, Patrick J Sinko

**Affiliations:** 1 Department of Pharmaceutics, Ernest Mario School of Pharmacy, Rutgers University, 160Frelinghuysen Road, Piscataway, New Jersey 08854-0789, USA; 2 Department of Molecular Genetics, Microbiology, and Immunology, Robert Wood Johnson Medical School, University of Medicine and Dentistry of New Jersey, Piscataway, New Jersey 08854, USA; 3 Cancer Institute of New Jersey, New Brunswick, New Jersey 08903-2681, USA

## Abstract

**Background:**

Current anti-AIDS therapeutic agents and treatment regimens can provide a dramatically improved quality of life for HIV-positive people, many of whom have no detectable viral load for prolonged periods of time. Despite this, curing AIDS remains an elusive goal, partially due to the occurrence of drug resistance. Since the development of resistance is linked to, among other things, fluctuating drug levels, our long-term goal has been to develop nanotechnology-based drug delivery systems that can improve therapy by more precisely controlling drug concentrations in target cells. The theme of the current study is to investigate the value of combining AIDS drugs and modifiers of cellular uptake into macromolecular conjugates having novel pharmacological properties.

**Results:**

Bioconjugates were prepared from different combinations of the approved drug, saquinavir, the antiviral agent, R.I.CK-Tat9, the polymeric carrier, poly(ethylene) glycol and the cell uptake enhancer, biotin. Anti-HIV activities were measured in MT-2 cells, an HTLV-1-transformed human lymphoid cell line, infected with HIV-1 strain Vbu 3, while parallel studies were performed in uninfected cells to determine cellular toxicity. For example, R.I.CK-Tat9 was 60 times more potent than L-Tat9 while the addition of biotin resulted in a prodrug that was 2850 times more potent than L-Tat9. Flow cytometry and confocal microscopy studies suggest that variations in intracellular uptake and intracellular localization, as well as synergistic inhibitory effects of SQV and Tat peptides, contributed to the unexpected and substantial differences in antiviral activity.

**Conclusion:**

Our results demonstrate that highly potent nanoscale multi-drug conjugates with low non-specific toxicity can be produced by combining moieties with anti-HIV agents for different targets onto macromolecules having improved delivery properties.

## Background

Most current anti-Acquired Immunodeficiency Syndrome (AIDS) drugs target two key enzymes in the human immunodeficiency virus-1 (HIV-1) replication cycle, reverse transcriptase and protease. While the remarkable efficacy of protease and reverse transcriptase inhibitor combinations for the treatment of HIV-1 infection has been clearly established *in vitro *and in the clinic, not even a single AIDS patient has ever been cured. Accordingly, new anti-HIV drug candidates having alternate mechanisms of action are under investigation. For example, ALX40-4C [1,2] blocks viral coreceptor CXCR4 and TAK-779 [3] blocks coreceptor CCR5. T-20 [4,5] and T-1249 [6-8] inhibit virus-cell fusion by binding to the viral envelope glycoprotein gp-41. Tat antagonists [9,10] interrupt viral transcription. NCp7 inhibitors [11] hamper viral assembly and budding. To date, combination pharmacotherapy remains the most effective strategy for reducing viral loads in HIV-infected patients. However, given the variety of new chemical entities under development, combination therapies hold even greater future promise.

A major impediment to successful anti-HIV-1 therapy is the emergence of drug resistant strains harboring mutations in genes encoding these viral enzymes [12]. Factors that are known or expected to contribute to the failure of highly active antiretroviral therapy (HAART) include pre-existing resistance [13], low and fluctuating drug concentrations due to poor drug absorption or patient non-compliance[6,14,15], and the presence of viral reservoirs and sanctuary sites [16]. Other mechanisms of resistance are becoming increasingly recognized in AIDS therapy. For example, drug-induced biopharmaceutical "resistance" (i.e., multidrug resistance), an established concept in cancer pharmacotherapy [17,18], occurs when the upregulation of cell efflux transporter activity results in lower cellular exposure and decreased drug efficacy. Therefore, the ability to control blood and cellular drug concentrations is critical for managing the emergence of classical viral and multidrug resistance.

**Figure 1:**
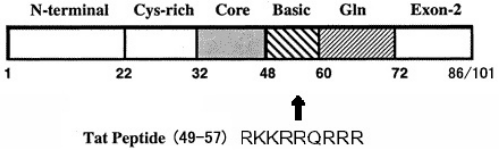
Schematic representation of Tat peptide, the basic domain in the viral Tat protein containing residue 49–57.

Recent successes with HIV peptide fusion inhibitors such as T20 (e.g., enfuvirtide and fuzeon) [8] suggest that small anti-HIV peptides can provide clinical utility complementing the antiviral activity of reverse transcriptase or protease inhibitors. However, many of these peptide drugs are poorly absorbed or are rapidly cleared from the body. HIV-1 encodes a small non-structural protein, Tat (trans-activator of transcription), which is essential for transcriptional activation of virally encoded genes. Viruses with deletion of the Tat-function are non-viable [19]. Efficient replication and gene expression of HIV-1 requires a specific interaction of the Tat viral protein with the trans-activation responsive element (TAR), a highly stable stem-loop RNA structure [20]. The interaction with TAR is mediated by a nine-amino acid basic domain (RKKRRQRRR, residues 49–57) of the Tat protein (Figure 1). This domain is essential for TAR RNA binding *in vivo *and is sufficient for TAR recognition *in vitro *[21]. A Tat-derived basic arginine-rich peptide alone binds TAR RNA with high affinity *in vitro *[9]. A peptidyl compound, N-acetyl- RKKRRQRRR-(biotin)-NH2, containing the 9-amino acid sequence of Tat protein basic domain, was shown to inhibit both Tat-TAR interaction *in vitro *and HIV-1 replication in cell culture [9]. In addition to the TAR RNA interaction, the basic domain in Tat has at least three other functional properties. It constitutes a nuclear/nucleolar localization signal [22,23]. The basic Tat peptide is also a prototypic cell penetrating peptide that can bring a cargo molecule across the plasma membrane. This was originally discovered when it was observed that Tat protein could freely enter cells [24]. Small Tat peptides derived from the basic domain have also been shown to inhibit HIV replication in cultured T-cells by interacting with the HIV CXCR4 co-receptor present on the surface of T cells, thereby blocking infection by T-tropic HIV-1 strains [25-27]. These peptides may also have translational effects [28].

Therefore, we have been investigating Tat peptides as therapeutic agents [9,28]. Considering the pleiotrophic effects of Tat domain peptides, it is not clear whether the delivery of these peptides to extracellular or intracellular targets or both is important for their antiviral effect. Furthermore, since these Tat peptides have cell penetrating activity [29], they can also potentially be used to enhance the cellular uptake of an appended drug[30]. However, Tat peptides have certain disadvantages, such as high systemic clearance due to *in vivo *degradation, non-specific binding to other biological components, and rapid renal clearance due to their low molecular weight and positive charges [31]. A variety of different strategies have improved biopharmaceutical properties of peptide drugs. In our studies, we have utilized retro-inverso (RI) peptides and macromolecular PEG conjugates to overcome the many biopharmaceutical challenges faced by Tat peptides. R.I.CK (retro-inverso-D-cysteine-lysine)-Tat9, N-acetyl-ckrrrqrrkkr-NH_2_, consists of D-amino acids assembled in the reverse order of the natural L-amino acid Tat9 peptide, N-acetyl-RKKRRQRRR-NH2. Thus, R.I.CK-Tat9 has a similar shape and charge distribution to the natural L-amino acid peptide but is more stable to proteases and retains pharmacological activity [32]. PEGylation has been shown to be one of the most successful techniques for improving the pharmacokinetic and pharmacodynamic properties of peptide drugs by increasing stability and reducing renal clearance and protein binding [33].

The second agent used in this study, saquinavir (SQV) was the first HIV-protease inhibitor approved by the U.S. Food and Drug Administration. Its structure mimics the phenylalanine-proline cleavage sequence at positions 167 and 168 of the HIV gag-pol polyprotein [34]. Thus, SQV prevents cleavage of gag and gag-pol protein precursors by HIV protease in acutely and chronically infected cells, arresting maturation and blocking nascent virions from becoming infectious [35]. However, therapeutic use of SQV suffers from problems of low absorptive and high secretory permeability, bioconversion to inactive metabolites, and poor solubility [36,37]. The oral bioavailability of SQV in clinical formulations is low and/or variable with limited penetration into the lymphatic and central nervous systems (CNS) [38,39]. While its low and variable bioavailability is primarily attributed to metabolism by cytochrome P-450 3A, recent results published by our group [40] and others [41] suggest that multiple membrane transporters may also contribute significantly to the delivery problems of SQV.

In a previous report [42], we showed that the activity of SQV prodrug conjugates was reduced when SQV was conjugated to PEG_3.4 K_, compared to the maximal achievable antiviral efficacy. But activity was restored by the addition of R.I.CK-Tat9 to the conjugate (EC_50 _= 15 nM). However, the mechanism of enhancement for the SQV-PEG_3.4 K_-R.I.CK-Tat9 conjugates was not clearly established since, in addition to targeting intracellular TAR, Tat possesses cell penetrating properties [29,30] that may promote conjugate uptake into the cell and/or it may exert anti-HIV-1 activity by means of cell surface binding to CXCR-4 receptors [25-27]. In the current study, the preclinical *in vitro *effectiveness of a small peptidic Tat antagonist, R.I.CK-Tat9, alone or in combination with saquinavir on multifunctional poly(ethylene glycol) (PEG)-based bioconjugates is demonstrated. Furthermore, the mechanism of enhanced activity of the SQV-PEG_3.4 K_-R.I.CK-Tat9 conjugates was addressed by flow cytometry and confocal microscopy. The current results suggest that the increased anti-HIV activity of SQV-PEG_3.4 K_-R.I.CK-Tat9 is due to the enhanced intracellular uptake and synergistic inhibitory effects of SQV on HIV protease and Tat peptides on both CXCR4 co-receptor interaction and/or HIV-1 transcriptional activation.

## Results

### Synthesis of R.I.CK-Tat9 and SQV conjugates

**Figure 2:**
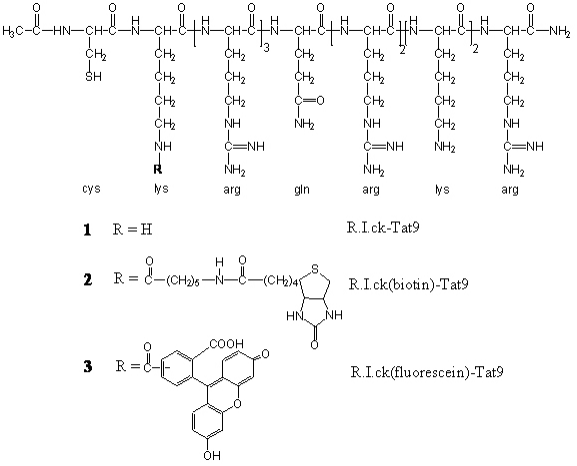
Schematic representation of R.I.CK-Tat9 and its derivatives.

**Figure 3:**
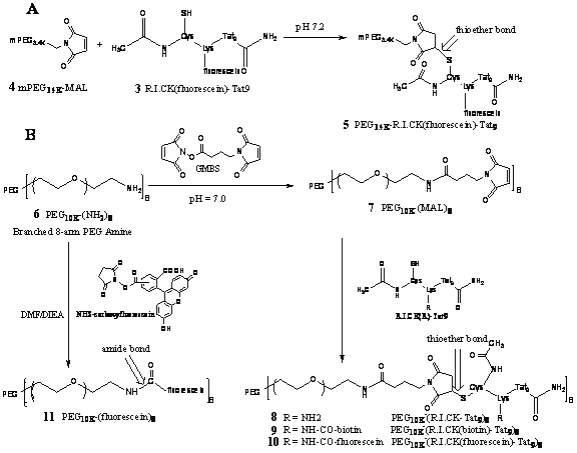
Synthetic scheme of Tat-PEG bioconjugates with single (3A) or multiple copies (3B) of R.I.CK-Tat9 and fluorescein-labelled control PEG lacking Tat peptides.

**Figure 4:**
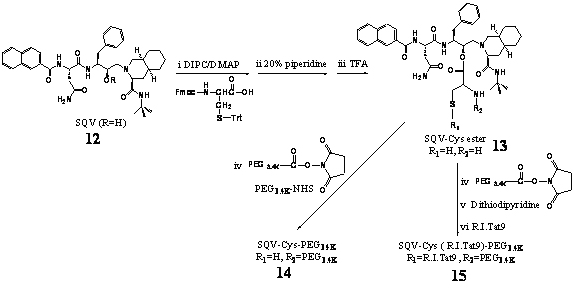
Synthetic scheme of Tat-SQV bioconjugates (i) 3 equivalents Fmoc-Cys(S-Trt)-COOH in CH_2_Cl_2 _with DIPC/DMAP; (ii) 20% piperidine in CH_2_Cl_2 _; (iii) TFA/CH_2_Cl_2 _(1:1); (iv) 2 equivalents Fmoc-PEG_3.4 K_-NHS in CH_2_Cl_2 _with DIEA; (v) 2 equivalents 2,2'-Dithiodipyridine in DMSO; (vi) 2 equivalents. R.I.CK-Tat9 in DMSO.

A series of R.I.CK-Tat9 and SQV bioconjugates was synthesized and characterized. The peptides, L-Tat9, R.I.CK-Tat9, R.I.CK(biotin)-Tat9, and R.I.CK(ε-carboxyfluorescein)-Tat9 (Figure 2) were synthesized, purified and their structures were confirmed by electrospray ionization mass spectrometry (ESI-MS). For R.I.CK-Tat9 PEG bioconjugates, the thiol group of the cysteine residue at the N-terminus of R.I.CK-Tat9 or its derivatives was linked to the maleimide group on mPEG-MAL (Figure 3A) or amino groups of branched 8-arm PEG_10 K_-(NH_2_)_8 _through a stable thioether bond using a heterobifunctional cross-linker N-maleimidobutyryloxysuccinimide ester (GMBS) (Figure 3B). As a control, the eight amino groups of 8-arm PEG amine were reacted with carboxyfluorescein-NHS and yielded PEG_10 K_-(ε-carboxyfluorescein)_8 _lacking Tat peptides. These bioconjugates were purified using size-exclusion chromatography on a Sephacryl S-100 column. The formation of each bioconjugate was confirmed by MALDI-TOF mass spectrometry and the concentration of each bioconjugates was determined by quantitative amino acid analysis. The overall design for all SQV conjugates was to link the various components using covalent bonds that varied in their stability properties (Figure 4). A biodegradable ester bond was made between the hydroxyl group of SQV and the carboxyl group of Cys. The esterification of the hydroxyl of SQV was confirmed by ESI-MS and ^1^H and ^13^C NMR. The thiol group of Cys was used to attach R.I.CK-Tat9 and its derivatives via a reducible disulfide bond, while the amino group of Cys was used to attach PEG_3.4 k _via a more stable amide bond. The thiol group of the cysteine in PEGylated form of SQV-Cys-ester was activated with 2,2'-dithiodipyridine. Then, disulfide bond formation resulted from addition of R.I.CK-Tat9 to the activated PEGylated form of SQV [SQV-Cys(PEG_3.4 k_)(TP)], giving SQV-Cys(PEG_3.4 k_)(R.I.CK-Tat9), in 65% yield after gel permeation purification. Mass spectrometry using MALDI-TOF of these products demonstrated peaks at the expected molecular weights.

### Stability studies

**Figure 5:**
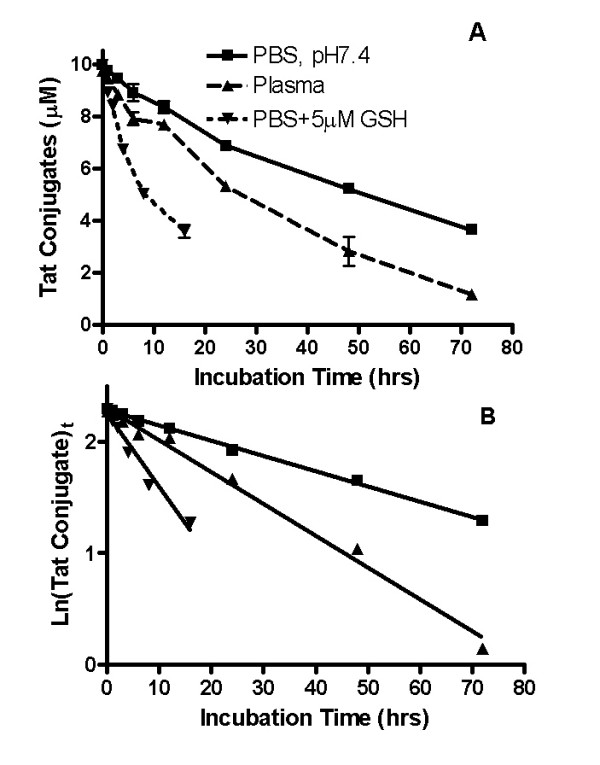
**Panel A: **Release of R.I.CK(fluorescein)-Tat9 from PEG_10 K_-[(R.I.CK(fluorescein)-Tat9]_8 _in PBS (pH 7.4) (■), in plasma (▲) or PBS (pH 7.4) with 5μM GSH (▼) at 37°C respectively, using fluorescence detection at excitation wavelength 485 nm and emission wavelength 535 nm. The concentrations of the bioconjugates were determined from fluorescence calibration curves that were established in the same media. All measurements were done in duplicates. **Panel B: **Plot of ln(bioconjugate)_t _versus incubation time (t) of the PEG- [R.I.CK(fluorescein)-Tat9]_8 _in PBS (pH 7.4) (■), in plasma (▲) or PBS (pH 7.4) with 5 μM GSH (▼) at 37°C respectively. The rate constant (k) is the slope of this linear plot. The half-lives (t_1/2_) for the thioether bond cleavage were calculated using the relation t_1/2 _= 0.693/k.

The stability of the covalent linkages attaching pharmacophores to the conjugates was determined. R.I.CK-Tat9 was linked to PEG_10 K _using a relatively stable thioether bond. The stability of this bond was assessed by incubating PEG10 K-[R.I.CK(fluorescein)-Tat9]_8 _bioconjugates in PBS (pH 7.4), spiked plasma or PBS (pH 7.4) with 5 μM reduced glutathione (GSH) at 37°C. Aliquots were withdrawn at different time points and centrifuged at 14,000 × g for 90 min with a 10 kDa cut-off Microcon™ filter. The conjugated R.I.CK(fluorescein)-Tat9 was retained in the filter as retentates and separated from the cleaved free R.I.CK(fluorescein)-Tat9 that passed through the filter. Thereafter, the fluorescence of conjugated R.I.CK(fluorescein)-Tat9 was measured using a Tecan fluorescence microplate reader with an excitation wavelength at 485 nm and an emission wavelength at 535 nm. This method was used rather than measuring fluorescence in the flow-through, since the concentration of cleaved R.I.CK(fluorescein)-Tat9 could not be quantified due a high degree of adsorption caused by its positive charges (unpublished data). The calibration curves of fluorescein-labeled R.I.CK-Tat9 in PBS (pH 7.4) and in plasma were linear with correlation coefficients of 0.9993 and 0.9997, respectively. The concentration of the bioconjugates decreased with time (Figure 5). Plots of ln [(bioconjugate)_t_] against incubation time (t) were linear within the concentration range studied indicating that cleavage occurs by a first order process (Figure 5). The PEG_10 k_-[(R.I.CK(fluorescein)-Tat9]_8 _bioconjugate showed a longer half-life (t_1/2 _= 50.6 h) in PBS (pH 7.4) than in plasma (t_1/2 _of 24.4 h). The half-life of this bioconjugate decreased significantly from 50.6 h to 10.5 h in the presence of 5 μM GSH in PBS (pH 7.4).

SQV was linked to R.I.CK-Tat9 through a SQV-Cys ester bond. The stability of the ester was evaluated in PBS at pH 7.4 and in spiked plasma, measured at 37°C using a recently developed fluorogenic protease assay for free SQV [42]. The release of active SQV was observed with half-lives of 14 h and 0.9 h in PBS at pH 7.4 and in spiked plasma, respectively [42].

### Biological activity

**Figure 6:**
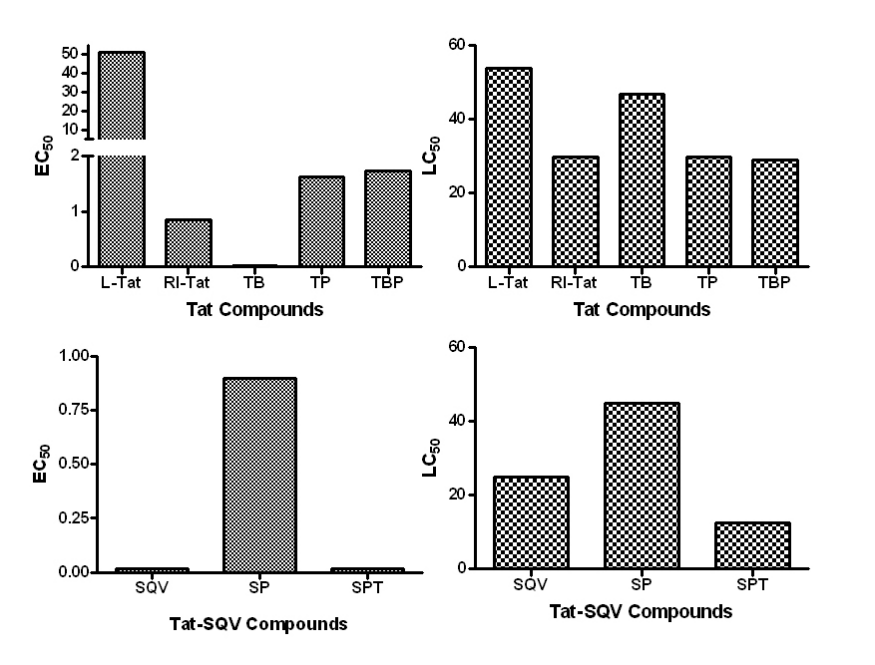
Representative data from MTT assays showing the anti-HIV activity (EC_50_) of Tat and SQV compounds using MT-2 cells infected with HIV-1 strain Vbu 3 at 0.01 MOI. Cytotoxiciy (LC_50_) was determined usjng uninfected cells. [TB: R.I.CK(biotin)-Tat9, TP: PEG_10k_-(R.I.CK-Tat9)_8_, TBP: PEG_10 K_-(R.I.CK(biotin)-Tat9)_8_, SQV: saquinavir, SP: SQV-Cys- PEG_3.4 K_, SPT: SQV- PEG_3.4 K_-R.I.CK-Tat9]

**Figure 7:**
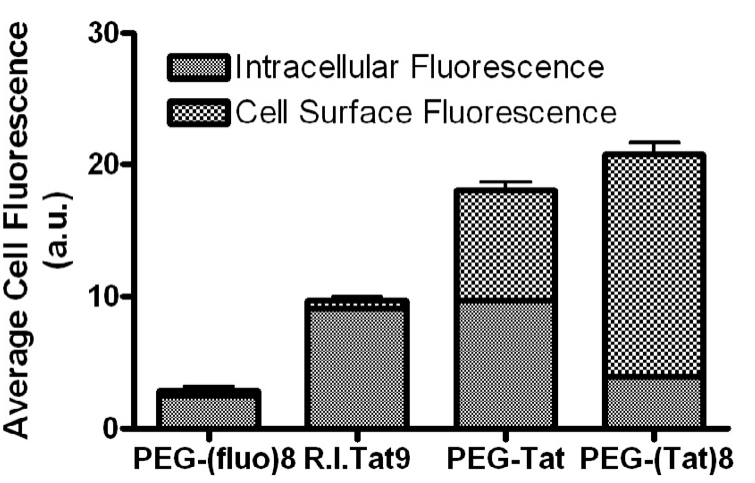
Quantitative data from flow cytometry showing the intracellular fluorescence and cell surface bound fluorescence of fluorescein-labeled PEG_10 K _[PEG-(fluo)_8_], R.I.CK-Tat9 (R.I. Tat9), PEG_3.4 K_-R.I.CK-Tat9 (PEG-Tat), and PEG_10 K_-(R.I.CK-Tat9)_8 _[PEG-(Tat)8] after 24 hrs incubation with MT-2 cells at 37°C for 24 hrs. The total cell associated fluorescence was quantified by flow cytometry. The intracellular fluorescence was measured after the cell surface-bound fluorescence was quenched by 0.2 mg/ml trypan blue at pH 5.8. The cell surface bound fluorescence is the difference between the total cell associated fluorescence and the intracellular fluorescence.

**Figure 8:**
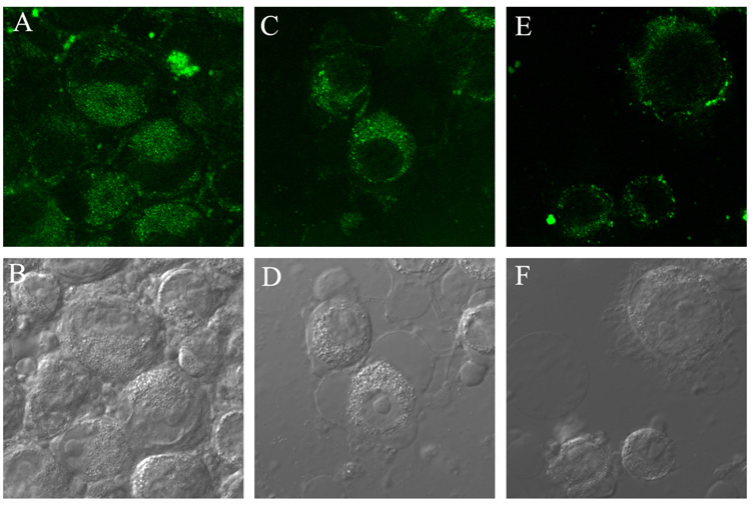
Confocal microscopic images of suspended MT-2 cells incubated with R.I.CK(fluorescein)-Tat9 (A, B), PEG_3.4 K_-R.I.CK(fluorescein)-Tat9 (C, D) and PEG_10 K_- [R.I.CK(fluorescein)-Tat9]_8 _(E, F) for 24 hours (all 1μM relative to Tat9). (A, C and E show fluorescence images while B, D and F are light images generated by differential interference contrast (DIC) of the same fields. All focal planes are through the middle of the cells. A and C show bright intracellular fluorescence, while E shows primarily cell surface bound fluorescence.

The anti-HIV activity of each bioconjugate derived from R.I.CK-Tat9 and SQV was evaluated *in vitro *in HIV-infected MT-2 cells (Figure 6) utilizing an established antiviral assay [42]. The L-form of Tat9 showed weak anti-HIV activity (EC_50 _= 51.3 μM) while the retro-inverso form of Tat peptide showed much stronger anti-HIV activity (EC_50 _= 0.85 μM). Biotin appended to the R.I.CK-Tat9 peptide greatly enhanced the activity of the peptide (EC_50 _= 0.018 μM), possibly due to the increased cellular uptake (~ 30-fold) conferred by biotin [43]. The conjugation with a 10 kDa branched PEG might be expected to enhance the stability of the peptide by protecting it from attack by peptidases, even though the unnatural D-amino acids in the R.I. peptides may already confer ample protease-resistance. We found that R.I.CK-Tat9 is released from PEG_10 k_-(R.I.CK-Tat9)_8 _conjugates very slowly (t_1/2 _= 50.6 h) and that most of the fluorescence labeled PEG_10k_-(R.I.CK-Tat9)_8 _conjugate remained bound to the MT-2 cell surface rather than being internalized (Figures 7 &8). Thus, the PEG conjugates of R.I.CK-Tat9 and R.I.CK(biotin)-Tat9 both displayed similar antiviral activity with EC_50 _of 1.47 μM and 1.5 μM, respectively, which was weaker than that of the non-PEGylated forms (Figure 6 and Table 1). This result suggests that the intracellular inhibitory effect of Tat peptide may be quantitatively more important than the extracellular blocking of HIV infection previously described for Tat-based peptides, although targeting cell surface receptors might still be an important secondary mechanism of viral inhibition by either biotinylated or non-biotinylated peptides.

**Table 1:** Anti-HIV activity (EC_50_) and cytotoxicity(LC_50_) data of R.I.CK-Tat9 based bioconjugates in MT-2 cell culture infected with HIV-1 strain LAV-Vbu3 (MOI of 0.01)^*a*^.

Compound	Number	EC_50 _(μM)	R^*b*^	LC_50 _(μM)	Therapeutic Index (LC_50_/EC_50_)
L-Tat9	-	51.3	-	53.8	1
R.I.CK-Tat9	1	0.85	-	29.8	35
R.I.CK(biotin)-Tat9	2	0.018	0.02	46.8	2600
PEG_10 K_-(R.I.CK-Tat9)_8_	8	1.47	1.7	29.1	20
PEG_10 K_-(R.I.CK(biotin)-Tat9)_8_	9	1.50	1.8	29.7	20
SQV(MeSO_3_H) (control)	12	0.015	-	25	1667
SQV-Cys-PEG_3.4 K_	14	0.90	60	4.5	50
SQV-Cys(R.I.CK-Tat9)-PEG_3.4 K_	15	0.015	1	12.5	833

^*a *^R is the ratio of the EC_50 _of the bioconjugate to that of its parent drugs R.I.CK-Tat9 or SQV.

^*b *^Several assays of each compound were done with variations in the virus stock, dosage and days of incubation.

The activity of SQV in drug conjugates compared to the maximal achievable antiviral efficacy of free SQV (EC_50 _= 15 nM) was reduced with addition of PEG_3.4 k _alone (EC_50 _= 900 nM), but restored with the addition of R.I.CK-Tat9 to the SQV-PEG_3.4 k _conjugate (EC_50 _= 0.015 μM), the same *in vitro *potency as free SQV.

The cytotoxicities of the R.I.CK-Tat9 and SQV bioconjugates were measured by incubating non-infected MT-2 cells in the presence of different concentrations of the bioconjugates for 5 days at 37°C. The cytotoxicity (LC_50_) of all the tested bioconjugates was in the low micromolar range (12.5 – 46.8 μM). The L-form of Tat9 showed the poorest therapeutic index with essentially equivalent EC_50 _and LC_50_. In contrast, a number of the multi-component bioconjugate molecules such as R.I.CK(biotin)-Tat9 and SQV-PEG_3.4 k_-R.I.CK-Tat9 exhibited very favorable therapeutic indices of 2600 and 833, respectively. The ratios of LC_50_/EC_50_(i.e., *in vitro *therapeutic index) are shown in Table 1.

### Flow cytometry and confocal microscopy

Flow cytometry (Figure 7) showed that MT2 cells incubated with fluorescein-labeled control PEG lacking Tat peptides had a low fluorescence and no cell-associated fluorescence by fluorescence microscopy (data not shown), indicating PEG did not bind or enter the cells. In contrast, cells incubated with 1 μM (concentrations of conjugates containing Tat9 are indicated in Tat9 equivalents) R.I.CK(fluorescein)-Tat9, PEG_3.4 k_-R.I.CK(fluorescein)-Tat9, or PEG_10 k_- [R.I.CK(fluorescein)-Tat9]_8 _had significant amounts of total cell-associated fluorescence, with cells incubated with PEG_3.4 k_-R.I.CK(fluorescein)-Tat9 and PEG_10 k_- [R.I.CK(fluorescein)-Tat9]_8 _having twice as much fluorescence as cells incubated with R.I.CK(fluorescein)-Tat9. When the cell surface-bound fluorescence was quenched with trypan blue, 93.8 % of the total cell-associated fluorescence was intracellular in cells incubated with R.I.CK(fluorescein)-Tat9, 53.6 % of total in cells incubated with PEG_3.4 k_-R.I.CK(fluorescein)-Tat9, and only 19 % in cells incubated with PEG_10 k_- [(R.I.CK(fluorescein)-Tat9]_8_. Since it is known that arginine-rich peptides bind to CXCR4, this suggests that multivalent Tat9 binding to CXCR4 on the cell surface impedes conjugate internalization.

Confocal microscopy studies (Figure 8) showed that in cells incubated with 1 μM R.I.CK(fluorescein)-Tat9 or PEG_3.4 k_-R.I.CK(fluorescein)-Tat9, there was significantly higher fluorescence intracellularly than on the cell surface. On the other hand, cells incubated with PEG_10 k_- [R.I.CK(fluorescein)-Tat9]_8 _showed primarily cell surface bound fluorescence. Note that in Figure 8, only a middle section of the cells is presented for each compound and the nucleus-cytosol boundaries of some cells can be discerned in the DIC (differential interference contrast) images. This result is consistent with the results from flow cytometry.

**Figure 9:**
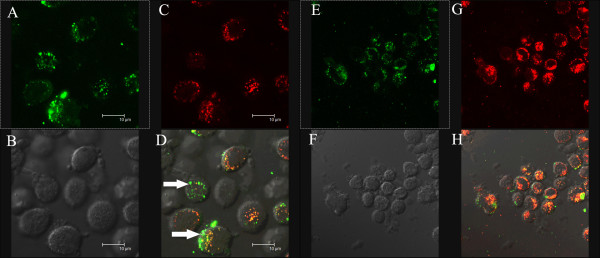
Confocal microscopic images of suspended MT2 cells incubated with R.I.CK(fluorescein)-Tat9 (A-D) or PEG_3.4 K_-R.I.CK(fluorescein)-Tat9 (E-H) in the presence of endocytosis marker rhodamine-dextran at 37°C for 24 hours. A and E show fluorescein fluorescence (green), B and F are DIC images, C and G show rhodamine-dextran (red), and D and H show overlaying of the green and red images. Colocalization of two dyes (orange-yellowin the overlay images) implies endosomal uptake of PEG_3.4 K_-R.I.CK-Tat9. Cells incubated with R.I.CK(fluorescein)-Tat9 showed some faint green fluorescence that was not co-localized with the fluid phase endocytosis marker (arrows in Figure 9D), suggesting some cytosolic localization.

Since the targets of both SQV (HIV-1 protease) and one of the Tat targets (HIV-1 mRNA TAR region) are in the cytosol/nucleus compartment and since flow cytometry and the confocal data shown in Figure 8 do not distinguish between the cytosol/nucleus and endosome compartments for the intracellular fluorescence, we incubated cells with either R.I.CK(fluorescein)-Tat9 or PEG_3.4 k_-R.I.CK(fluorescein)-Tat9 and a fluid phase endocytosis marker, tetramethylrhodamine-labeled dextran (10 kDa). The results (Figure 9) showed that at a relatively high concentration (7μM) R.I.CK(fluorescein)-Tat9 or PEG_3.4 k_-R.I.CK(fluorescein)-Tat9 (green) were mainly co-localized with the fluid phase endocytosis marker rhodamine-dextran (red) in punctate dots (orange/yellow in the merged panels, Figure 9D &9H), suggesting predominant endosomal location. Only in cells incubated with R.I.CK(fluorescein)-Tat9, was there some faint green fluorescence that was not co-localized with the fluid phase endocytosis marker (arrows in Figure 9D), suggesting some cytosolic location.

Overall, the confocal data are consistent with the conclusion from flow cytometry analysis that the majority of R.I.CK-Tat9 is within cells, the majority of PEG_10 k_-(R.I.CK-Tat9)_8 _is on the cell surface, and PEG_3.4 k_-R.I.CK-Tat9 is roughly equally distributed between cell surface and intracellular locales. The confocal data further suggest that after exposure to 1μM conjugate, intracellular R.I.CK-Tat9 and PEG_3.4 k_-R.I.CK-Tat9 are all predominantly within endosomes. Since the cytosol/nucleus compartment accounts for the vast majority of cellular volume, any R.I.CK-Tat9 and PEG_3.4 k_-R.I.CK-Tat9 molecules that escaped from endosome, or entered cytosol directly from outside cell, would be diluted. As a result, the detection of cytosolic fluorescence is not sensitive and we cannot rule out the presence in cytosol/nucleus compartment of a fraction of total intracellular R.I.CK-Tat9 and PEG_3.4 k_-R.I.CK-Tat9. Therefore, the potent anti-HIV-1 activity of SQV-PEG_3.4 k_-R.I.CK-Tat9 due to addition of the R.I.CK-Tat9 moiety could be attributable to a variety of factors including the inhibitory effect of Tat9 peptide on viral interaction with co-receptor CXCR4 and/or on HIV-1 transcriptional activation, either of which might be synergistic with SQV inhibition of HIV protease.

## Discussion

While recent advances in anti-AIDS therapeutics have resulted in the introduction of more potent drugs, a cure for HIV infection remains an elusive goal. Many factors contribute to the inability of current therapeutic regimens to cure HIV infection. However, central to the problem is the variability of drug concentrations in the blood and target tissues resulting from poor patient adherence to complicated regimens, the inability of these potent agents to selectively target infected tissues, and the poor penetration or retention of drugs in reservoir and sanctuary sites. It is becoming more obvious that better drug delivery and targeting technologies are required to increase total body persistence, target cell exposure and retention of these potent therapeutic agents. In the current study, we have produced and tested the first of a series of nanoscale drug delivery vehicles in order to better target HIV infected cells and possibly to provide novel modes of action.

The present report describes the design, synthesis and initial characterization of a series of PEG-based bioconjugates in order to achieve maximum therapeutic payload of Tat peptide, to explore multi-drug delivery on one bioconjugate and to determine the potential role of Tat in the cellular uptake and HIV inhibition by the conjugates. These bioconjugates were designed (i) to carry multiple copies of the R.I.CK-Tat9 drug linked by stable thioether bonds to 8-arm poly(ethylene) glycol (PEG), (ii) to carry multiple drugs such as SQV for combination therapy, (iii) to have an extended biological and chemical half-life, (iv) to selectively release appended drug molecules inside the cell because of the differential in reducing capacity between blood and the internal cell environment and finally, (v) to enhance cellular uptake of the drug and bioconjugate through the use of uptake enhancing moieties like biotin attached to the R.I.CK-Tat9. These modifications were designed to increase the therapeutic peptide's bioavailability, biodistribution and delivery into HIV sanctuary sites of both the therapeutic Tat peptide and appended drugs, exemplified here by SQV.

The current study is aimed at improving the therapeutic potential of a Tat-antagonistic compound R.I.CK-Tat9. It is an analog of the nine amino acid sequence of the TAR-binding basic domain of Tat protein in which the direction (polarity) of the amino acid sequence is reversed and the chirality of each amino acid residue is inverted from L to D. Retro-inverso analog peptides are expected to have shapes and charge distributions of their side chains similar to the natural L-amino acid peptides, but are highly resistant to proteolysis [44,45]. Furthermore, Wender et. al. found that the L-, D- and R.I. forms of Tat9 showed similar cellular uptake in serum-free medium, while in the presence of serum the D-form was modestly more active and the R.I. form much more active than the L-form, indicating the likely role of proteolysis in limiting the activity of natural peptides [46]. The current results show that R.I.CK-Tat9 had a 60-fold higher anti-HIV activity than L-Tat9, consistent with the enhanced stability and/or increased intracellular availability of R.I.CK-Tat9.

Choudhury et al. showed that Tat9-C(biotin) with S-biotinylation of the cysteine residue was taken up 30-fold more efficiently by Jurkat cells than was unbiotinylated Tat9-C (3% versus 0.1%, respectively) [9]. This was attributed to increased hydrophobic interactions with the plasma membrane [43] and it was hypothesized that biotinylation would result in enhanced inhibiton of transactivating activity by the biotinylated compound. The current results confirm this hypothesis since the biotinylated RI-Tat9 was 47 times more potent than the RI-Tat9 and was approximately as potent as SQV in inhibiting HIV-1.

The central hypothesis of the current study is that improved delivery would result in an enhancement in the pharmacological properties of Tat inhibitors, in particular R.I.CK-Tat9. Thus, it was anticipated that PEGylation of R.I.CK-Tat9 would enhance the pharmacological properties *in vivo *for more effective delivery. The PEG residues were chosen to avoid *in vivo *binding of R.I.CK-Tat9 to plasma proteins and rapid elimination from the blood [47]. Thus, PEGylation provides a way to increase the stability and body persistence of the R.I.CK-Tat9, which could result higher *in vivo *activity. However, the PEG conjugates reduced the antiviral activity of R.I.CK-Tat9 or R.I.CK(biotin)-Tat9 in cell culture experiments (Table 1). The thioether bonds, used in the linkage between R.I.CK-Tat9 or R.I.CK(biotin)-Tat9 and PEG were very stable, insuring that Tat was not released from the conjugate. These results suggest the quantitatively more important antiviral effect of R.I.CK-Tat9 depends upon its release from PEG, presumably reflecting a requirement for entry into infected cells.

An oligocationic peptide compound (ALX40-4C), designed to mimic the basic domain of the HIV-1 Tat [2], was also found to interfere with viral entry through the inhibition of the chemokine receptor CXCR4 on the host cell membrane [1]. The blocking of viral entry resulted in a more potent response than the inhibition of transactivation by that compound [2]. To delineate whether the anti-viral mechanism of the R.I.CK-Tat9 conjugates is by inhibition of transactivation or by blocking of cell surface co-receptor, R.I.CK-Tat9 was labelled with the fluorescence tag carboxyfluorescein-NHS and conjugated to PEG_3.4 K _and PEG_10 K _for stability and uptake mechanism studies of the final conjugates. Flow cytometry showed 93.8%, 53.6%, and 19.0% of total cell-associated R.I.CK(fluorescein)-Tat9, PEG_3.4 K_-R.I.CK(fluorescein)-Tat9, and PEG_10 K_- [R.I.CK(fluorescein)-Tat9]_8_, respectively, were within the cells. In contrast, the control fluorescein labelled PEG lacking the Tat peptide, PEG_10 K_-(fluorescein)_8_, showed little cell association by flow cytometry and no cell surface binding by fluorescence microscopy (data not shown). The confocal microscopy studies showed that cells incubated with 1μM R.I.CK(fluorescein)-Tat9 or PEG_3.4 K_-R.I.CK(fluorescein)-Tat9 showed significant higher intracellular fluorescence, while cells incubated with PEG_10 K_- [R.I.CK(fluorescein)-Tat9]_8 _showed primarily cell surface-associated fluorescence. These results suggested that the observed anti-HIV activity of the uncleavable PEG_10 K_-(R.I.CK-Tat9)_8 _conjugates is a result of the binding of the conjugate to cell surface CXCR4 receptor, which is consistent with observations of other groups [1,2]. However, the reduced potency of the conjugates relative to free R.I.CK-Tat9 suggests that this peptide may have more anti-HIV-1 activity at intracellular sites than at the cell surface. These results support the model that the most potent mechanism of action of this peptide agent is inhibition of the transcriptional effects of the viral Tat protein.

While expected to be a stronger binder to CXCR4, PEG_10 K_-(R.I.CK-Tat9)_8 _is less promising than PEG_3.4 K_-R.I.CK-Tat9 in intracellular targeting. The intracellular space consists of two topological compartments – the cytosol/nucleus and all membrane-bound organelles including endosomes and lysosomes. HIV-1 protease and reverse transcriptase, the cellular targets of the majority of current anti-HIV-1 drugs, are located in the cytosol, whereas the TAR region of all HIV-1 mRNA transcripts, the cellular target of both conjugates and their released R.I.CK-Tat9, is located in the nucleus. Compared to PEG_3.4 K_-R.I.CK-Tat9, less PEG_10 K_-(R.I.CK-Tat9)_8 _is internalized. Significant portions of both internalized conjugates are within endosomes. Unless they escape, endosome-confined conjugates and their drug-carrying derivatives cannot reach these cellular targets. At present, there is some evidence in the literature for the endosomal escape of Tat peptide-conjugates or fusion proteins. We recently discovered a possible pH-dependent endosomal escape mechanism that could operate at the mildly acidic pH of 6.5 of early endosomes (to be published). Once they escape from endosomes, both conjugates and their released R.I.CK-Tat9 must enter the nucleus in order to meet their TAR target. The cytosol and the nucleus are continuous through nuclear pores, which have a functional diameter of about 40 nm. This pore size roughly equals the effective size of a linear PEG molecule of 20 kDa [48], implying that the 25 kDa PEG_10 K_-(R.I.CK-Tat9)_8 _might not be able to enter the nucleus while the 5 kDa PEG_3.4 K_-R.I.CK-Tat9 might.

In this study, we used uninfected MT2 cells that are transformed CD4^+ ^T cells, which are capable of supporting HIV-1 replication. Depending on a particular anti-HIV-1 agent tested and the MOI (multiplicity of infection) used, MT2 cells can serve as a useful model for HIV-1 infected or uninfected T cells *in vivo*. Critical to current HAART regimens is that both infected and uninfected T cells should be adequately loaded with anti-HIV-1 drugs at all times. In the infected cells, the drugs prevent the production of mature, infectious viruses. In the uninfected cells, the drugs play a prophylactic role, ensuring abortive infection or replication whenever infection occurs. Constant new HIV-1 infection must take place as both HIV-1 and virus-replicating T cells have short *in vivo *half-life yet the virus keeps evolving and cessation of HAART inevitably leads to viral load rebounding. We now know that during all stages of HIV disease CD4^+ ^T cell depletion occurs predominantly in the gut [49].

Certain aspects of the performance of PEG conjugates (e.g., plasma persistence and protein binding) can only be studied *in vivo *and are not addressed in the current study. The known *in vivo *advantages of PEGylation of various pharmacophores indicates the potential that PEG-based bioconjugates could display useful therapeutic properties of increased plasma half-life [50], lower cytotoxicity [51], and reduced protein binding, which could outweigh the observed reduction in efficacy seen in this study for some PEGylated drugs. This will require further future study in *in vivo *models and is beyond the scope of the current studies.

Multiple-drug cocktail regimens have been associated with the recent successes in improving the quality of life of patients with AIDS. However, delivery of drugs to the bloodstream rather than to many of the known sites of high viral replication may not insure that the maximal therapeutic effect will be obtained. A potential first step in achieving this maximal effect is to better control the exposure of infected cells to multiple therapeutic agents. In this proof-of-principle study, the prototypical protease inhibitor, SQV, was conjugated to PEG_3.4 K _and PEG_3.4 K_-R.I.CK-Tat9. The conjugation of PEG to SQV yielded a much less active prodrug conjugate SQV-Cys- PEG_3.4 K _with EC_50 _at 900 nM. The 60-fold lower activity of the conjugate compared to the parent drug could be due to the slow cleavage of the ester bond and/or low cell uptake of the conjugate. However, the addition of Tat to SQV-Cys- PEG_3.4 K _resulted in a conjugate with an EC_50 _of 0.015μM, the same *in vitro *potency as the free SQV. The increased activity of SQV-PEG_3.4 K_-R.I.CK-Tat9 may be attributable to a variety of factors including the enhanced intracellular uptake by the bifunctional conjugates containing both Tat peptide and SQV, the synergistic effects of SQV on HIV protease and Tat peptides on both HIV Tat-TAR binding and/or on cell surface receptor CXCR4. All of these results suggest that higher anti-HIV activity was attained mainly due to the enhanced intracellular uptake of SQV-PEG_3.4 K_-R.I.CK-Tat9 resulting in improved delivery of SQV. However, the synergistic effects of SQV on HIV protease and Tat peptides on both HIV TAR and/or CXCR4 are likely to contribute.

## Conclusion

In the current study, the preclinical *in vitro *effectiveness of a small peptidic Tat antagonist, R.I.CK-Tat9, alone or with saquinavir using PEG-based bioconjugates was demonstrated. While the PEG-linkage alone did not potentiate the activity of R.I.CK-Tat9, the addition of SQV to R.I.CK-Tat9-PEG bioconjugates significantly enhanced the anti-retroviral activity to the level comparable to free SQV. These bifunctional conjugates were more potent than the PEG conjugates with R.I.CK-Tat9 or SQV alone. These results demonstrate that the macromolecular bioconjugates could deliver drugs to multiple targets, bringing drug delivery systems down to the molecular level. Since *in vitro *studies of anti-HIV activity do not account for factors that would enhance *in vivo *potency (e.g., increased body persistence and decreased binding), it is quite possible that the advantages of SQV-PEG_3.4 K_R.I.CK-Tat9 conjugates over the parent drugs could become evident by *in vivo *testing. In addition, all of the bioconjugates demonstrated low cytotoxicity.

The modular approach for producing a targeted nanopharmaceutical delivery system that we have taken allows for appending different drug combinations or combinations of drugs and cellular uptake enhancing agents in order to maximize the therapeutic effect. Therefore PEG bioconjugates could constitute a powerful delivery system for either single drug administration or combination therapy. However further mechanistic studies are needed to optimize the structure (e.g. size, shape and other features) of the PEG bioconjugates. Additional studies are needed to assess the potential *in vivo *benefits (long half-life, lower clearance rate) of these conjugates.

## Methods

### Synthesis of R.I.CK-Tat9 and SQV conjugates

R.I.CK-Tat9 and its derivatives, compounds **1–3 **(Figure 2) were synthesized manually on a MBHA Rink amide resin (Novabiochem, La Jolla, CA) via Fmoc chemistry in the presence of coupling activating reagents, BOP (benzotriazol-1-yl-oxytris(dimethylamino)phosphonium hexafluorophosphate) and HOBt (*N*-hydroxybenzotriazole) (Sigma-Aldrich, St. Louis, MO). The ε-Dde (1-(4,4-dimethyl-2,6-dioxocyclohex-1-ylidene)ethyl) protecting group (in the N-terminal lysine residue) was selectively removed using 2 % hydrazine in DMF (dimethylformamide) and reacted to attach the appended groups biotin-NHS or carboxyfluorescein-NHS (Sigma-Aldrich, St. Louis, MO) on solid support. The peptides were then acetylated, cleaved and purified on a Vydac C_18 _column (10 μm, 2.2 × 25 cm, Vydac, Hesperia, CA) with detection at 220 nm; fluorescence of labelled peptides was detected at 535 nm (emission) and 485 nm (excitation). The purified products were lyophilized and confirmed by electrospray ionization mass spectra(ESI-MS).

In the synthesis of R.I.CK-Tat9-PEG bioconjugates (Figure 3), R.I.CK(fluorescein)-Tat9 was conjugated to mPEG-MAL by the reaction of the thiol group of R.I.CK(fluorescein)-Tat9 with the maleimide group on mPEG-MAL (**4**), which formed a stable thioether bond between PEG and R.I.CK(fluorescein)-Tat9. The eight amino groups of PEG_10 K_-(NH_2_)_8 _(MW 10 kDa, 8-arm branched, Nektar Therapeutics, Huntsville, AL) (**6**) were first activated with three-fold molar excess of the heterobifunctional cross-linker, N-maleimidobutyryloxysuccinimide ester **(GMBS) **(Pierce Biotechnology, Rockford, IL), in DMF to form a maleimide activated PEG (**7**). The reaction was stirred overnight at room temperature. The product was precipitated with cold ether and dried under vacuum to yield the solid PEGylated product. The GMBS linker essentially converts a primary amino group to a maleimide group that can react with a thiol group to form a stable thioether bond. This activated intermediate **7 **was reacted with a three-fold molar excess of **1**, **2 **and **3**, respectively, with coupling reagents HOBt (4-fold molar excess) and BOP (3-fold molar excess) in DMF. DIEA (diisopropylethylamine, 1 % v/v) was added to adjust to neutral pH. For control, the eight amino groups of 8-arm PEG amine were reacted with carboxyfluorescein-NHS in the presence of 1%DIEA in DMF to yield **11 **PEG_10 K_-(fluorescein)_8 _lacking Tat peptides. The products were recrystallized from cold ether and dried under vacuum overnight. These bioconjugates **8–11 **were purified using size-exclusion chromatography using a Sephacryl S-100 column (Amersham, Piscataway, NJ) in 0.1 M PBS, pH 7.4, with detection at 220 nm (for **8 **and **9**). For **10**, fluorescence was detected at 535 nm (emission) and 485 nm (excitation). The formation of the bioconjugates (**8–11**) was confirmed by MALDI-TOF mass spectrometry and the concentrations of bioconjugates were determined by amino acid analysis.

In the synthesis of the SQV conjugates (Figure 4), the active hydroxyl function of SQV (**12**, extracted from Inverase, Roche) was esterified with Fmoc-Cys(S-Trt)-COOH using DIPC (1,3-diisopropylcarbodiimide)/DMAP [4-(dimethylamino)pyridine] as coupling reagent. SQV-Cys ester (**13**) was obtained with 82% yield after Fmoc removal with piperidine, followed by TFA(trifluoroacetic acid) -deprotection of the Trt (trityl) group. The esterification of the hydroxyl of SQV after silica gel purification was confirmed by ESI-MS and ^1^H and ^13^C NMR. ESI-MS (m/z): 774.5 (M+H)^+^; 796.5 (M+Na)^+^. PEGylation of **13 **was carried out using PEG_3.4 k_-NHS in the presence of DIEA/DCM to give **14**. This product was purified using gel permeation chromatography (Sephadex LH-20 column in DMF, 239 nm) resulting in 70 % yield. The formation of **14 **was confirmed by MALDI-TOF (m/z (%) 3837.6) and ^1^H and ^13^C NMR. The thiol group of the cysteine in PEGylated form of SQV-Cys-ester **(14) **was then activated with 2,2^'^-dithiodipyridine. Addition of **1 **to the activated PEGylated form of SQV-Cys-ester (**14**) gave **15 **with 65% yield after gel permeation purification. Mass spectrometry of these products demonstrated peaks at the expected molecular weights using MALDI-TOF (m/z (%) 5447.6.

### Release kinetics of R.I.CK-Tat9 by cleavage of thioether bond

The stability of fluorescein-labeled PEG_10 k_-(R.I.CK-Tat9)_8 _bioconjugates was investigated in PBS (pH 7.4) and rabbit plasma, at 37°C. The stability of the bioconjugates was also investigated in PBS (pH 7.4) at 37°C by treating them with 5 μM glutathione (GSH) (Sigma-Aldrich, St. Louis, MO), a physiologically relevant reducing reagent that is responsible for intracellular reductive environment inside cells. Initially, different concentrations (0.01 – 10.0 μM) of R.I.CK(fluorescein)-Tat9 were dissolved separately in PBS (pH 7.4) and in plasma and their fluorescence measured by Tecan fluorescence microplate reader (excitation at 485 nm, emission at 535 nm) to obtain calibration curves in PBS (pH 7.4) and in plasma. Thereafter, the bioconjugate solutions were incubated separately in PBS (pH 7.4) and in spiked rabbit plasma at 37°C, along with GSH treated bioconjugates in PBS (pH 7.4) at 37°C. Aliquots were withdrawn at different time points and centrifuged at 14,000 × g for 90 min with a Microcon™ filter (molecular weight cut-off = 10,000 Da) (Amicon Inc., Beverly, MA). The drug moiety cleaved from the bioconjugate during the incubation passes through the filter whereas the drug moiety that remains linked to the PEG carrier is retained. [Note that a PEG polymer of 3,400 Da behaves as a >10,000 Da peptide on ultrafiltration.] The retentates resulting from the different incubation time points were withdrawn and subjected to fluorescence detection. Each measurement was done in duplicate. The concentrations of the bioconjugates were determined from a fluorescence calibration curve that was established in the same media. The rate constant (k) was obtained from the linear plot of ln(bioconjugate)_t _versus incubation time, t (h), where [bioconjugate]_t _= concentration of bioconjugate at different incubation time, t, (Figure 5). The half-life (t_1/2_) of the cleavage of thioether bond from the bioconjugates was calculated from the relation t_1/2 _= 0.693/k where k is the slope of the linear plot.

### Release kinetics of SQV by cleavage of ester bond

A fluorogenic protease inhibition assay was used to measure the hydrolysis kinetics of SQV-Cys ester which was the common intermediate for all the synthesized R.I.CK-Tat9-SQV bioconjugates. The chemical stability of this ester was determined in PBS at pH 7.4 and in spiked plasma both measured at 37°C as reported earlier [42].

### Antiviral assays

The in vitro anti-HIV activity of PEG conjugates was determined by a MTT-based HIV-1 susceptibility assay reported previously [42] using MT-2 cells, an HTLV-1-transformed human T-cell leukemia cell line, infected with the HIV-1 strain LAV-Vbu3. The cytotoxicity of the conjugates was evaluated in parallel. The MT-2 cells were grown in RPMI 1640 DM (Dutch modification) medium supplemented with 20% FBS (fetal bovine serum), 1% w/v pen-strep, and 1% w/v L-glutamine and maintained at 37°C in 5% CO_2 _in an incubator. The cultured MT-2 cells were diluted to 2 × 10^5 ^cells/mL and infected at a multiplicity of infection (MOI) of 0.01 (1 viral particle per 100 cells), causing the death of 90% of the cells 5 days later. The tested conjugates were diluted with RPMI 1640 medium and were added to the cultured MT-2 cells after viral infection. Each conjugate was tested in triplicate for its antiviral activity. Cell viability was measured by the colorimetric MTT test at 540 nm, which is directly proportional to the number of living cells. The conjugate EC_50 _and LC_50 _values were determined from the curves of the percentage of viral cell killing and cytotoxicity against compound concentration (Table 1, Figure 6).

### Confocal microscopy

MT2 cells were treated with fluorescein-labeled R.I.CK-Tat9 or its conjugates for 24 hours. Where indicated, a fluid phase endocytosis marker, tetramethylrhodamine-Dextran/10 kDa (Invitrogen/Molecular Probe), was used at 0.25 mg/ml in co-incubation with a fluorescein-labeled compound. All images were taken of live cells on a Leica TCS SP2 Spectral Confocal Microscope using the XYZ mode and 0.25 micrometer per section. The fluorescence wavelength windows for different dyes were well separated to ensure no breach-through from one dye to another.

### Flow cytometry

MT-2 cells were grown to 2 days post-confluency and aliquots of 2 × 10^6 ^cells were washed briefly and incubated in 96-well microplates with 1 μM PEG_10 K_, R.I.CK(fluorescein)-Tat9, PEG_3.4 K_-R.I.CK(fluorescein)-Tat9 and PEG_10 K_- [R.I.CK(fluorescein)-Tat9]_8 _bioconjugate for 24 hours. Trypan blue staining was used for quenching of cell surface bound fluorochrome emission. After the 24 hours incubation with tested conjugates, the medium was immediately removed from the wells. Part of the cells were washed and resuspended in 0.02 M sodium acetate buffer (pH 5.8). The remaining cells were suspended in sodium acetate buffer containing 0.2 mg/ml trypan blue. After 20s, the cells were washed twice and resuspended in the sodium acetate buffer. The total cell associated fluorescence was then analyzed by flow cytometry using a Coulter EPICS PROFILE equipped with a 25 mW argon laser. For each analysis, 10,000 to 20,000 events were accumulated. The total cell associated fluorescence was the cell associated fluorescence of cells without quenching by trypan blue. The intracellular fluorescence was the cell associated fluorescence of cells quenched by trypan blue. The cell surface bound fluorescence is the difference between the total cell associated fluorescence and the intracellular fluorescence.

## Competing interests

The author(s) declare that they have no competing interests.

## Authors' contributions

**LW**: Performed synthetic and quality control chemistry, stability, flow cytometry, confocal microscopy studies, interpretation of results and participated in drafting the manuscript.**XZ**: Performed confocal microscopy and participated in study and conjugate design and drafting the manuscript.**SG**: Performed synthetic and quality control chemistry and participated in drafting the manuscript.**SP**: Performed synthetic and quality control chemistry.**OD**: Performed HIV and general toxicity studies.**MJL**: Participated in study and conjugate design and critically reviewing manuscript.**ABR**: Participated in study design and supervised HIV and toxicity studies and critically reviewing manuscript.**SS**: Participated in conjugate and study design and interpretation of results.**PJS**: Participated in conjugate and study design and interpretation of results and drafting the manuscript.
